# Unprecedented Density and Persistence of Feral Honey Bees in Urban Environments of a Large SE-European City (Belgrade, Serbia) †

**DOI:** 10.3390/insects12121127

**Published:** 2021-12-16

**Authors:** Jovana Bila Dubaić, Slađan Simonović, Milan Plećaš, Ljubiša Stanisavljević, Slobodan Davidović, Marija Tanasković, Aleksandar Ćetković

**Affiliations:** 1 Faculty of Biology, University of Belgrade, Studentski Trg 16, 11000 Belgrade, Serbia; jovanabila@bio.bg.ac.rs (J.B.D.); mplecas@bio.bg.ac.rs (M.P.); acetkov@bio.bg.ac.rs (A.Ć.); 2 SOS Mobile Team for Rescue and Removal of Honey Bee Swarms and Colonies, Koste Glavinića 12, 11000 Belgrade, Serbia; bioplodplus@ptt.rs; 3 Department of Genetics of Populations and Ecogenotoxicology, Institute for Biological Research “Siniša Stanković”—National Institute of the Republic of Serbia, University of Belgrade, Bulevar Despota Stefana 142, 11060 Belgrade, Serbia; slobodan.davidovic@ibiss.bg.ac.rs (S.D.); marija.tanaskovic@ibiss.bg.ac.rs (M.T.)

**Keywords:** *Apis mellifera*, citizen science, feral honey bees, natural selection, unmanaged honey bees, urban environment

## Abstract

**Simple Summary:**

The western honey bee, a pollinator species that is essential for modern agriculture and food production sustainability, is under various anthropogenic pressures. In the last few decades, these have led to serious worldwide problems concerning the health and stability of honey bee colonies. The importance of wild or feral honey bees has only recently been recognized, as these populations are crucial for research on the processes that enable their survival under such pressures. Here, we present a case of an unmanaged free-living population of honey bees that, unlike in other known studies, live in a highly populated urban environment. This extraordinarily dense feral honey bee population, which is not directly associated with managed apiaries, provided us with a large dataset of various life history parameters that will considerably fill in the knowledge gaps on unmanaged colonies. Furthermore, we want to underline the importance of citizen science in the data collection process and suggest it as a suitable approach to study feral honey bees in urban landscapes. We believe that highly populated urban landscapes can support and reinforce feral honey bees and that similar citizen science projects should be set up in other urban areas or other countries.

**Abstract:**

It is assumed that wild honey bees have become largely extinct across Europe since the 1980s, following the introduction of exotic ectoparasitic mite (*Varroa*) and the associated spillover of various pathogens. However, several recent studies reported on unmanaged colonies that survived the *Varroa* mite infestation. Herewith, we present another case of unmanaged, free-living population of honey bees in SE Europe, a rare case of feral bees inhabiting a large and highly populated urban area: Belgrade, the capital of Serbia. We compiled a massive data-set derived from opportunistic citizen science (>1300 records) during the 2011–2017 period and investigated whether these honey bee colonies and the high incidence of swarms could be a result of a stable, self-sustaining feral population (i.e., not of regular inflow of swarms escaping from local managed apiaries), and discussed various explanations for its existence. We also present the possibilities and challenges associated with the detection and effective monitoring of feral/wild honey bees in urban settings, and the role of citizen science in such endeavors. Our results will underpin ongoing initiatives to better understand and support naturally selected resistance mechanisms against the *Varroa* mite, which should contribute to alleviating current threats and risks to global apiculture and food production security.

## 1. Introduction

The western honey bee, *Apis mellifera* Linnaeus, 1758, is recognized as the single most important pollinator species, vital to the success of modern agriculture and the stability of human food production [1,2,3,4,5], let alone the provisioning of honey and other bee-products. There is a worldwide concern regarding the trends of its populations, including various health issues and maintenance of sufficient pollination ecosystem service [3,6,7,8,9,10,11,12,13,14]. In the last two decades, numerous studies identified several factors that negatively affect honey bee populations and beekeeping [7,15,16,17]. Among other factors, the exotic ectoparasitic mite *Varroa destructor* (hereafter: *Varroa*) is considered the most significant threat to western honey bee in many parts of the world [18,19,20,21,22,23], largely contributing to widespread colony losses [24,25]. There is a widely accepted view that unmanaged (wild/feral) honey bee populations were completely eradicated in Europe since the 1980s, following the introduction and spread of *Varroa* and the associated spillover of various pathogens [26,27]. However, several studies reported that both feral and managed colonies can survive for an extended period despite of *Varroa* infestation, and without receiving any treatments, triggering wide scientific and public attention [28,29,30,31,32,33,34,35,36,37].

There is a general lack of knowledge regarding feral colonies, since the majority of research is focused only on managed honey bees [38,39,40]. Given that unmanaged honey bees are not receiving any chemical treatments against *Varroa* or other pests/pathogens, it is important to explain how they manage to survive. Successful cohabitation with *Varroa* is well known in closely related Asian species (*Apis cerana*), which co-evolved with this parasite [23]. Understanding the mechanisms behind these interactions might provide clues for improving the health status and fitness of managed *A. mellifera* colonies and securing perspectives for sustainable beekeeping [41,42,43,44]. In that respect, various studies focused on genetic differences between feral and managed colonies [31], or on more efficient hygienic and grooming behaviors [35,45] that might have evolved in colonies left untreated against parasites. Several studies suggested that differences of living conditions between free-living and managed honey bees (Table S5) may generate essentially different susceptibility to *Varroa* mite [36,46,47].

Finding wild/feral honey bees in nature is difficult and time consuming [38,48]. In areas where they exist (e.g., in the USA), feral colonies are scattered, mostly in woodland areas, usually high in the trees, and generally not easy to spot [38,48]. Potentially, urban environments could also be suitable for feral honey bees, due to ample nesting opportunities [49,50], the presence of diverse floral resources throughout the season [51,52,53], and lesser exposure to pesticide spraying [54]. However, there are only very few records of feral honey bees in urban environments [48,49,50].

Here, we present another case of an unmanaged, free-living population of honey bees. Unlike the previously reported cases, our study area is a large and highly populated urban environment, the city of Belgrade (capital of Serbia). Until several years ago, local scientists involved in various bee studies were generally unaware of the existence of abundant feral honey bees in and around Belgrade, while local beekeepers were not familiar with the importance of this phenomenon. Therefore, a ‘discovery’ of a huge data-set documenting numerous thriving colonies living completely without any kind of human interference or treatment came as a surprise. Data were obtained through the long-term activity of one of the authors (S.S.), a beekeeper then affiliated with the Belgrade Beekeepers Society (BBS). Specifically, S.S. was engaged in extensive communication with the Belgrade citizens who reported on honey bee colonies or swarms in their surroundings, mostly asking for their removal. Therefore, the compilation of the core data-set may be regarded as “incidental crowd sourced observations” also known as “opportunistic citizen science” [55]. It is well known that citizens’ help (e.g., the implementation of citizen science) can effectively overcome various difficulties in gathering information on biodiversity in urban environments [56,57].

The purpose of this study was to:

- present evidence of the extraordinary frequent incidence of unmanaged honey bee colonies throughout the wider Belgrade area (based on the occurrence data compiled for 2011–2017), and a similarly frequent incidence of swarms (probably many of them from unmanaged colonies);
- assess the basic parameters of the recorded occurrences (the features of documented nesting/swarming sites: type of substrate/microspace, height, neighbourhood or habitat type) and the parameters of the recording and reporting process (features of the compiled data-set, its limitations, peculiarities, and utility);
- analyze the patterns of distribution of free-living honey bee colonies and observed swarms across the different local urban and sub-urban/peri-urban settings of a large city, relative to the distribution of managed hives;
- evaluate the status of unmanaged honey bee occurrences in Belgrade, hypothesizing that they indicate the existence of a large, long-lasting and self-sustaining feral population;
- present the experiences (advantages, problems and perspectives) of the citizen science approach for detecting, assessing the status and monitoring of feral honey bees in urban environments.

## 2. Materials and Methods

### 2.1. Case Study Area

The city of Belgrade (44°49′ N 20°27′ E), the capital and the largest city in Serbia, is located at the confluence of two rivers, the Danube and the Sava. The municipal boundary of the city is roughly 35 × 36 square km, while the core administrative–urbanistic unit covers around 776 km^2^. Around 1.7 million people live within the total area of Belgrade, while up to 1.2 million people are located in the urban zone [58]. The climate is transitional between the temperate–continental and steppic regimes and its relief is spanning the altitude of 65–506 m.

### 2.2. Data Source

Data for this survey were gained through the compilation of the citizen requests for removal of honey bee colonies and/or swarms, mostly occurring near their homes or work place in the period 2011–2017. As Belgrade does not have an official utility service for this, all such requests have been redirected to the Belgrade Beekeepers Society (hereafter: BBS), who kept records and provided removal service (in that period operated by S.S.). Most records included the following information: date of request, location—address, category (swarm or colony), nesting/swarming site (type of substrate, etc.), contact information of observer, and intervention status. The intervention status refers to the personal notes of the visited site and of the removed swarm/colony by the beekeeper. He also kept notes on swarms that flew away on their own in which instances no intervention was required. Due to logistic and time constraints, especially in the swarming season, not all reported locations were visited. However, at most visited locations, successful removal or capture of swarms was conducted, which was noted in the original database. Sometimes, the removal of colonies was unsuccessful due to a lack of equipment to reach colonies in high places in the reported location.

### 2.3. Data Processing

**Figure 1:**
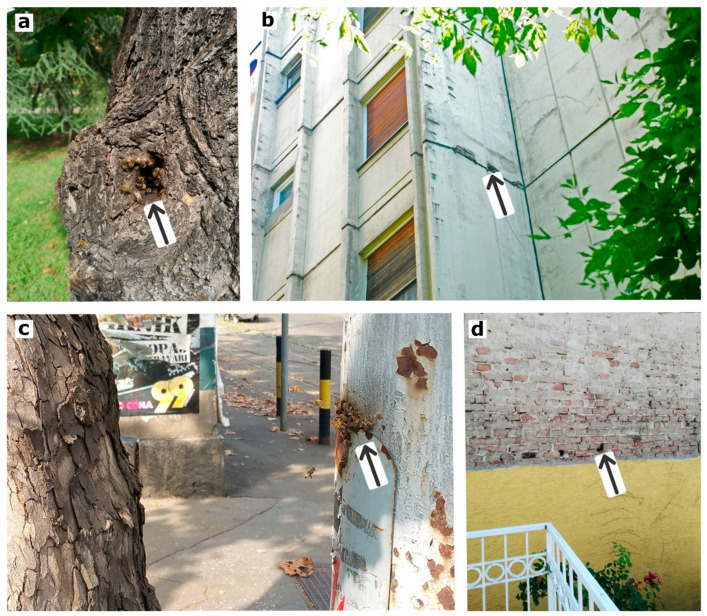
Some of the free-living honey bee colonies observed in Belgrade during 2019: (**a**) inside the hollow tree, (**b**) in the hollow space within a multi-story building floor, (**c**) inside the steel tubular electric pole, and (**d**) in a cavity of a damaged façade (showing propolis staining around the entrance).

During the studied period of seven years (2011–2017), more than 1700 removal requests were received. We performed several steps of refinement and cleaning of the initial data. Each removal record was first categorized into one of four categories: “bee colony”, “bee swarm”, “colony or swarm”, and “other”. The third category, “colony or swarm”, was created to account for incomplete reports that could not be resolved—when it was not possible to distinguish between colony and swarm, based on the information from the initial notes. The fourth category, “other”, contains reports of other forms of insects (mainly social wasps: yellowjackets and hornets), and they were excluded from the analysis. The next step was to detect duplicated records of swarms and colonies. When two (or more) records of swarms were reported on the same location and on the same date, or in the interval of one to two days, we regarded them as duplicates (i.e., the same occurrence reported by the different persons). Detecting duplicates in the category of honey bee colonies posed more difficulties. Even if the records originate on the same day and at the same location, there is no certainty whether it is one or more colonies involved. On some occasions the same colony is a nuisance to two or more neighboring apartments, and if both inhabitants filed a report, this makes one of the reports a duplicate. Additional difficulty may arise from the following situation: there were several reports of a honey bee colony all noted at the same location but in different years, which may relate to the same long-lasting colony reported by the same (or different) person year after year. To clarify situations like this, as well as to confirm and verify the rest of the data from our database, we carried out a process of validation. The validation process consisted of two steps: the first step was to conduct interviews with citizens who reported swarms/colonies, and the second step was to carry out a field study where possible (which was carried out in 2019) and gather information by observing reported locations (Figure 1).

During the interviews, our focus was to address the following: to ask about missing information, to describe what they can remember about swarms/colonies they reported, to inquire on the precise location of the hive/swarm, the height of the occurrence, and if they knew about the longevity of a colony they noticed. Furthermore, our intention was to see what happened after they reported it—are the bees still there? Did the beekeeper remove them? Was there a case of a newly established colony in the same cavity?

Original data rarely contained information about the longevity of reported colonies. During the validation process and interviews, we were particularly interested in this aspect. We were especially focused on reporters from the core of urban area, since occurrences within and nearby the tentative feral zone are considerably more relevant for the purpose of our study.

After each interview, the person was classified as “reliable” or “not reliable”, and therefore the information received was equally categorized. This thorough quality check was possible mainly because all interviews were conducted by one person (JBD), who spent considerable time working with this data set, first getting familiar with it, then reviewing each record and detecting duplicates, then mapping data (georeferencing locations), and finally conducting interviews with citizens. This allowed for an informed estimation of whether the person and resulting data were valuable or anecdotal (characterized by insufficient information or chaotic/contradictory statements, obvious lack of understanding of the object reported, etc.). We eliminated unreliable, duplicated, and incorrect data. The remaining records were mapped using the QGIS program [59].

We also mapped the presence of managed apiaries in the wider city area, based on the list of registered hives, personal communication with BBS, and recorded unregistered apiaries that were found during location inspections or other field surveys conducted in the study area.

For an informed analysis and better visualization of compiled data, we used the QGIS visualization tool—Heatmap (Kernel Density Estimation; hereafter: KDE)—to create density maps of reported colonies and swarms [59]. This analysis creates differently shaded surfaces, each shade representing a different density of the respective analyzed units. After a couple of trial runs, we used a search radius of 1000 m and 10 m px size since these parameterizations were found to be the best fitting for our data set.

In order to analyze the relationship between human population density and unmanaged honey bee colony or swarm occurrences, we performed point pattern analysis (Poisson point process model—ppm) [60,61]. We used the Belgrade official map of local communities (the lowest administrative unit for which standardized census data are available) as a spatial layer to define the analysis and population density of each local community in the form of a raster layer as a covariate. The reported occurrences of feral bees were defined as a planar point pattern (ppp). The analysis was done using the spatstat R package v.1.63 [60] within R v3.6.3 [62].

## 3. Results

**Figure 2:**
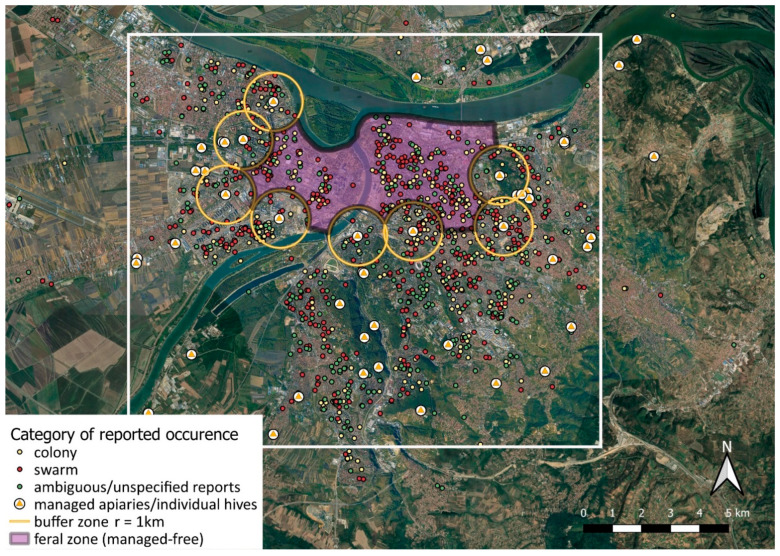
Distribution of georeferenced occurrences of unmanaged (potentially feral) honey bee colonies and swarms of unknown origin in the period of 2011–2017, within the wider Belgrade area (24 more remote locations from our data-set are not shown, being too widely scattered beyond the coverage of this map). Three categories of reported cases are shown combined but with differently colored circles (nesting colonies, swarms, and ambiguous or unspecified reports—the latter could be either a colony or a swarm; the separated distribution maps are available in Figure S1a–c). The locations of known managed apiaries (or individual hives) are also shown, with particular focus on those that surround the urban core area (each shown with a circular ‘buffer zone’ of r = 1 km). The urban core is presumed to harbor mostly the self-sustained feral bee colonies, and consequently, the swarms produced mostly by them; accordingly, we delimited a tentative ‘feral zone’ (or ‘managed-free zone’). The white rectangle (ca. 16.2 × 14.4 km) delimits the area shown in more detail in further maps. (This map is also available as a high-resolution image, upon request to J.B.D.).

For the period of seven successive seasons (2011–2017), we compiled a total of 1745 calls/reports (Table S1), of which 1495 were reporting on honey bees and 250 on other insects. After the removal of duplicated reports (124), our data-set includes 1371 unique reports (Table 1). Of those, for 56 reports we could not retrieve locations, hence 1315 reports were georeferenced (Figure 2; Figure S1). Unique reports comprise 460 colonies, 537 swarms, and 374 ambiguous/unspecified reports (occurrences that could represent either a colony or a swarm, but original evidence lacked details or contained contradictory elements).

**Figure 3:**
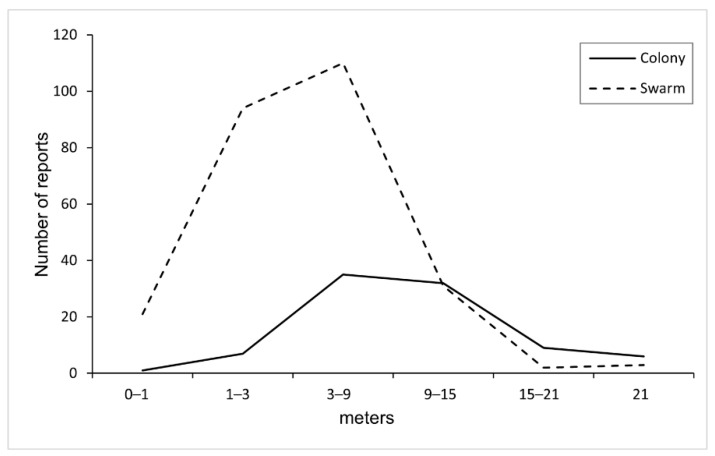
Height distribution of reported nesting/swarming sites of unmanaged honey bees (full line: colonies, dashed line: swarms).

**Figure 4:**
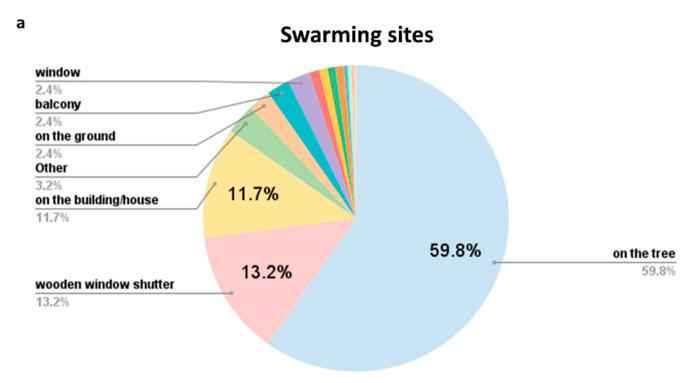

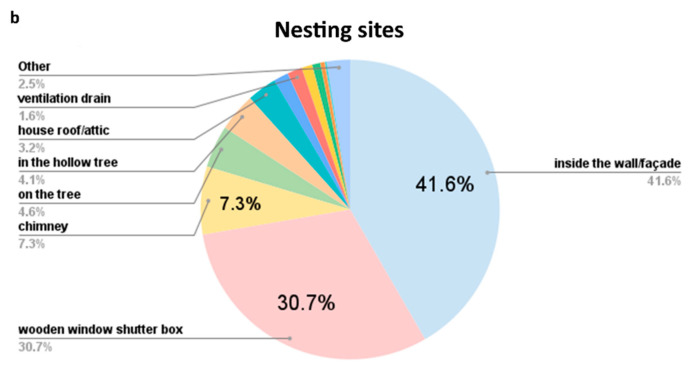
Distribution of records categorized by types of (**a**) swarming and (**b**) nesting sites.

Out of 1371 reports, information about height of the nesting/swarming site was available for 391 (Figure 3; Table S3), while information about the type of nesting/swarming site was known for 1195 reports (Figure 4; Table S4). Of these, around 85% colonies were recorded between 3 and 21 m, while 90% swarms were recorded between 1 and 15 m. Nesting/swarming site type was recorded for 437 of the reported colonies (95%) and for 463 of reported swarms (86%). The most common type of nesting cavity was inside the walls or various building/house façades (182 occurrences, ca. 42%), followed by the wooden shutter window box (134 occurrences, ca. 31%). The most common swarming situation was the hanging from a tree branch (277 occurrences, ca. 60%) (Figure 4).

**Figure 5:**
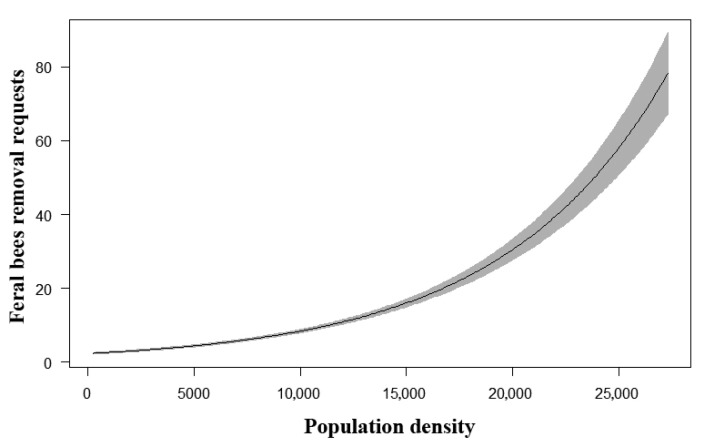
Relationship between human population density and the reports of unmanaged honey bees from the point process model. The gray zone represents a 95% confidence interval.

Analysis of the effect of human population density on reporting about unmanaged honey bee occurrences showed a strong positive relationship (Table 2, Figure 5). The overlapping distribution of human population density (by analyzed ‘local communities’ of Belgrade; data from 2011) and georeferenced occurrences of feral honey bee units is shown in Figure S2.

**Figure 6:**
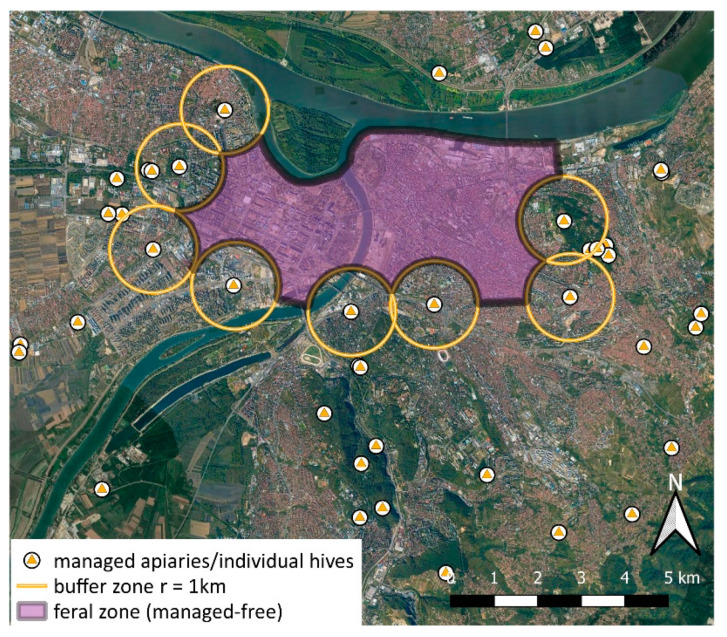
The central part of the study area, most intensively covered by citizens’ reports. Close-up view of the core feral/managed-free zone of Belgrade, and the locations of managed apiaries/hives which surround it (with their respective buffer zones).

Based on the presence and distance from managed apiaries (the circular buffer of 1 km), we defined a tentative ‘feral’ (=managed-free) zone in the core area of the city (Figure 6). Due to specific geographical features, the northern border of feral zone is represented by the Danube River, which is ca. 480–680 m wide in this section, hence represents a barrier to the potential influx of swarms from the north. 

**Figure 7:**
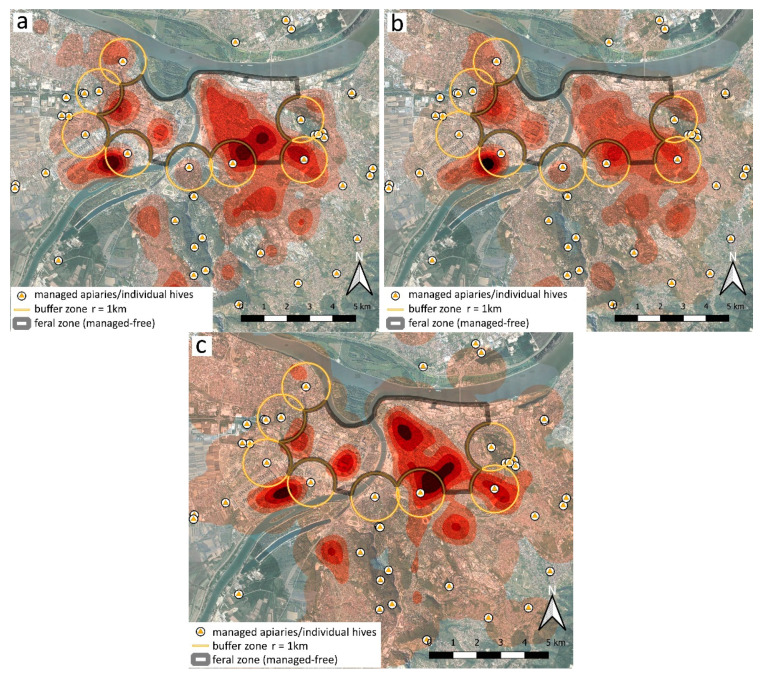
Kernel Density Estimation heatmaps: shading intensity represents the increasing density of respective occurrences (defined through six density zones for the search radius 1 km), based on summed reports for 2011–2017: (**a**) all categories, combined (min. 5.7, max. 43.8, zonal mid-point value range: 6.5–40.1); (**b**) colonies (min. 3.8, max. 26.6, zonal mid-point value range: 4.1–24.4); (**c**) swarms (min. 2.7, max. 18.1, zonal mid-point value range: 2.9–16.6).

We generated three separate KDE heatmaps: for all georeferenced reports of unmanaged honey bees (combined: the sum of all three categories), for the colonies, and for the swarms (Figure 7). Densities are shown as six color grades of the same intensity range, but the respective city areas with same color represent quite different maximal estimated values: ca. 18 for swarms, 27 for colonies, and 44 for all occurrences (the later include also the ambiguous records, which represent >27% of the whole data-set). The distribution of areas with highest density of different categories shows various levels of concordance.

**Figure 8:**
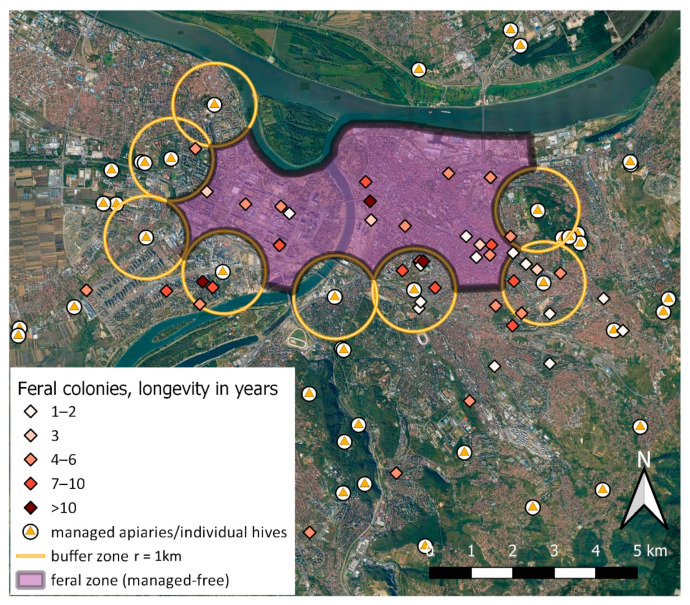
Distribution of feral colonies with documented activity potentially extending beyond the single season: evidence was principally based on repeated observations of the very same nesting space, obtained through reports/interviews with citizens and/or through personal checking by one of the authors (S.S.).

We managed to obtain more detailed and sufficiently reliable information for 78 nesting sites with tentative long-lasting continual activity of respective colonies (Table 3, Figure 8). Out of these, 18 colonies were from the interior of the feral zone, and further 22 from the neighboring area (within the buffer zone of ca. 2 km around feral zone, mostly within the respective buffer zones around centrally positioned managed hives/apiaries).

## 4. Discussion

For the context of our research, it is important to stress that urban beekeeping was almost a non-existent activity in most of the urban core of Belgrade (BBS, personal communication; the situation is slowly changing after 2018). Even at a moderate distance from central urban areas, managed apiaries are still relatively rare and scattered (Figure 6). Therefore, it seems quite unlikely that so many free-living colonies (and consequently most of the swarms) in these central areas are derived from regular swarm inflow from managed hives. Accordingly, we expect that >90% of the occurrences within the tentative feral zone might be essentially feral. 

This zone represents the urban core of residential and business activities, characterized by dense urban infrastructure, high human population density, and economic activities, accompanied by appropriate traffic dynamics. In contrast, the periphery of Belgrade is characterized with more widespread presence of beekeepers and apiaries of various types. Many of the reported swarms or unmanaged colonies in that area are likely not truly feral, but recently derived from managed hives. Between the feral zone and the periphery, a type of ‘transitional area’ may be operatively defined. The transition is related to two principal patterns: type of settlements is gradually changing (from densely urbanized to nearly rural/agricultural), while the incidence of beekeeping is growing (but largely varying in management efficiency). Accordingly, we expect that as many as 50–75% of the reported swarms and colonies in this ‘transition zone’ might also be feral. Much lower values are expected across the peripheral areas, but also the incidence of unmanaged honey bee reporting was generally more sparse.

The distribution pattern of reported occurrences, presented with HDE heatmaps from three data-subsets, shows reasonable concordance between the most densely inhabited city areas (Figure 7). Obviously, large extent of central urban area and few peripheral tracts are quite densely populated with free-living honey bees, with a few distinctive “cores of incidence” being situated in or nearby our feral (managed-free) zone. Two prominent cores are seemingly related to the two locations with managed apiaries (known, from anecdotal beekeepers sources, for excessive poorly managed swarming in past times). However, it is noteworthy that none of these cores are exactly centered at respective apiary, but rather offsetted in different directions, indicating the likely influence of habitat suitability and attractiveness. Indeed, the ‘western feral core’ is effectively settled within the particular type of relatively uniform neighbourhood, with multi-storey buildings of peculiar construction and uniform age, known empirically for numerous suitable nesting cavities. The ‘eastern feral core’ shows a ‘tendency of spreading’ towards the most urbanized downtown area of Belgrade. This is the largest continuous while also almost evenly populated part of the city; it is most distantly situated from the managed hives around the feral zone. Noteworthy, two of the three cases of longest-living colonies (or alternatively, the longest active nesting site—see also later) are practically centered in these two ‘feral cores’ (Figure 8). Therefore, it is possible that, even if initially established and ‘boosted’ from the managed hives, these two cores may now serve—for more than a decade—as ‘secondary source’ of numerous feral swarms, which continually populate the preferred urban sectors in a self-sustainable manner.

This possibility, that many unmanaged colonies (and respective share of swarms) in the Belgrade urban area represent an essentially feral and persistent population has been further corroborated by the results of a separate genetic study. Within the project SERBHIWE [63], S.D. and M.T. analysed the genetic variability of feral and managed honey bee colonies in the Belgrade area by molecular genetics methods; a brief summary outcomes are presented herewith. They sampled feral bees from 40 colonies selected from within our data-set and used two types of genetic markers [64]: uniparentally inherited mitochondrial DNA (mtDNA) and biparentally inherited microsatellites from autosomal loci. The analysis of the mtDNA *tRNA^leu^-cox2* intergenic region demonstrated a similar pattern of genetic variability for both types of sampled colonies (feral and managed), while the presence of rare haplotypes was detected in the mtDNA gene pool that could be found in only one group, either feral or managed. The analysis of 14 microsatellites loci showed that the feral honey bee colonies possess greater genetic diversity compared to the managed ones, and the assessed relatedness showed that on average, a feral honey bee colony is more related to other feral honey bee colonies than managed ones. These results suggest that swarming from managed apiaries is not the only reason for existence of such a great number of feral honey bee colonies in Belgrade. In other words, the abundant feral population may not be regarded as primarily or predominantly derived from the contemporary managed hives. Overall, molecular genetic analysis suggests the existence of a strong and genetically diverse population of feral honey bees in Belgrade. Hypothetically, this urban population of free-living honey bees may have existed in continuum from the period before the introduction of *Varroa* mite. Greater genetic diversity could have contributed to natural selection for improved tolerance against parasites and pathogens, and hence their capability for successful survival despite these pressures.

Available chemical treatments against *Varroa* mite do not provide a long-term solution (due to the development of resistance to pesticides), while some are even harmful to bees [65,66]. Moreover, any kind of treatment may inhibit natural selection pressures preventing coevolution between parasite and host, thus being counterproductive [33,43,67,68]. In uncontrolled/wild settings natural selection will lead toward disease-resistant bees and less virulent forms of pathogens [69,70]. Generally, wild populations of domesticated plants and animals are important reservoirs of genetic diversity [71] and this applies to wild and feral honey bees as well [27,39,72]. However, beekeepers are generally not willing to support such a course for economic reasons—expected high colony losses in initial period [43,73], but also the possibility of losing some of the desirable traits of managed bees [23]. For decades, breeding programs selected traits like greater honey production, less aggressive temperament, a lower tendency for swarming, high fecundity of queen, etc. [23,46,74,75]. In fact, honey bees that are in balance with new parasite are economically more beneficial to manage in the long run [43,70]. Recently, selection programs started to include disease resistance as one of the wanted traits of honey bees [74,75]. Hence, the spontaneously established *Varroa*-resistant honey bee population, like the one thriving in Belgrade, could be of great importance for future breeding programs in Europe.

Wild honey bees in natural habitats prefer to inhabit cavities in tree trunks [76]. Several studies reported on feral honey bees being opportunistic regarding the cavity types used for nesting in urban environments [49,50]. Our study provided extensive further details about the nesting site features of feral honey bees. The most commonly observed type of cavity was inside the walls or building/house façades (41.6%), followed by the cavities of wooden window shutter box (30.7%). Both types of cavities are closely associated with human living spaces that are heated/insulated, hence energetically beneficial for the colony, especially during the winter [77]. The advantage of certain human-associated cavity types for honey bees has also been emphasized in a recent study from Ireland [78].

Information on the longevity of individual colonies is a direct indicator of *Varroa* resistance level. In our data set, there were several colonies with exceptional duration. However, a seemingly continuous bee activity around a certain cavity does not necessarily imply the continual usage and persistence of the same colony (or its direct descendants). Instead, a colony may die off, but a swarm from another colony may move in, and this change in cavity status (from inactive to active) is called “turnover” [79]. Turnovers are easily missed; hence, the colony may be categorized in a wrong longevity class. Therefore, the longevity of free-living colonies has to be checked frequently and regularly [80]. Based on our experience from Belgrade (from 2019), for reliable longevity estimate, monitoring must be conducted as frequently as 10–14 days. If the long-active bee cavity cannot be monitored appropriately, such situations are better termed ‘extended cavity occupancy’ than colony longevity.

In addition to presenting a huge data set, this paper also highlights the importance and suitability of the citizen science (CS) approach for studying feral honey bees in urban settings. Finding feral honey bee colonies is difficult and time-consuming [38,48], particularly in the cities. The presence of a nuisance species, e.g., the new pest, will be primarily detected by the members of the public [81]. Honey bees are generally considered as beneficial insects, but they can represent a nuisance if inhabiting undesirable locations—too close to human activities. In addition to ‘ordinary’ citizens, beekeepers and urban pest control agencies can be a particularly important source of data [50,82]. Certainly, participatory research has its shortcomings, e.g., requires a longer process of validation, as in case of our data-set. Communicating with a substantial number of people can be challenging for several reasons; various problems which we encountered are summarized in Table S2.

Our study falls into the category of incidental citizen science, known also as “opportunistic citizen science” [55,83,84], which is linked with several biases (i.e., reporting, taxonomical, observational and geographical biases). Honey bees are widely known species, distinctive, and easily recognizable; hence, taxonomic bias in our data set is presumably not significant. Monitoring of easily recognizable species, such as honey bees, is generally feasible with CS projects [85], but untrained people often have difficulties distinguishing between swarm and colony. Honey bees are perceived in several different ways by the public [50], ranging from aversion and fear [86], to fondness and generally positive attitude towards them [87,88]. Consequently, people had strong reasons not to ignore, but to report occurrences of honey bees. Therefore, reporting bias is probably not significant to our study. Observational bias also could not be greatly present in our data since all observations of honey bee swarms and colonies were detected in close proximity to homes of people who reported it, not as a result of a search effort. On the other hand, we noticed significant geographical bias—there is evident lack of data of the city’s industrial and peripheral areas. Lack of data from industrial, non-populated areas, occurred simply because colonies and swarms are more likely to be noticed in densely populated residential areas of the city (proximity to human daily lives, high frequency of human activity), rather than in unpopulated or sparsely populated parts [48,49]. There were practically no reports of bee colonies or swarms in unpopulated areas because there was no one who could make a report [89]. On the other hand, lack of data from the periphery is due to the people living there would likely deal with the problem themselves. On the contrary, in the urban core, colonies are more likely to be reported because citizens who live there are less likely to have beekeeping experience [90]. The presence of swarms and colonies on the edges of the city, where managed apiaries are frequent, could originate from the swarm escapes from managed hives [76].

The successful existence of a dense, self-sustaining feral honey bee population in Belgrade is further promoted by certain socio-economic circumstances. For example, problems with unregulated jurisdiction (responsibilities) of public services in Belgrade, combined with a shortage of specialised employees and funding, make public utility companies inefficient and inadequate to respond to citizens’ requests for removing honey bee colonies/swarms; accordingly, many of them remain untreated. Furthermore, the extended poor state of Serbia’s economy (for many decades now) reflects on poor maintenance of many buildings (which makes urban areas rich in suitable cavities). For similar reasons, the majority of people cannot afford to pay for removal to private services (by professional beekeepers), again contributing to many colonies/swarms being left undisturbed. All these circumstances made Belgrade almost the perfect environment for the establishment and persistence of a large free-living honey bee population. There are also less specific favourable aspects, common to many large urban areas [91], but they certainly contributed to the overall good conditions for wild bees: variable and long-lasting floral resources, and decreased exposure to pesticides (relative to dominating agricultural systems, etc.).

In our case, the existence of a strong and self-sustaining feral honey bee population confirms that urban environments can be highly favourable for this species. However, a combination of circumstances which enabled or enhanced its establishment might have been locally or regionally context-specific, in several aspects. Nonetheless, it could be rewarding to investigate, following our experiences, if other cities in Europe (particularly larger cities in SE Europe) might also harbor the overlooked free-living honey bees.

## Figures and Tables

**Table 1:** All uniquely reported observations of unmanaged honey bees (i.e., without duplicated reports of the same units), grouped into: colonies, swarms, and ambiguous or unspecified units (could be either a colony or a swarm). Sums include 56 reports that could not be georeferenced.

Year	Colonies	Swarms	Ambiguous/Unspecified	Total Honey Bees
2011	157	95	-	252
2012	116	78	-	194
2013	34	38	38	110
2014	54	115	109	278
2015	33	56	78	167
2016	44	95	110	249
2017	22	60	39	121
∑	460	537	374	1371

**Table 2:** Estimated regression coefficients and their standard errors from a point process model showing the relationship between human population density and reports of unmanaged honey bees.

Variable	Coefficient (SE)	95% CI	z Value	z Test
Intercept	0.842421 (0.046938)	0.7504–0.9344	17.947	<0.05
Population density	0.000128 (0.000004)	0.000121–0.000136	34.384	<0.05

**Table 3:** Tentative longevity of feral colonies: number of nesting sites with documented honey bee activity potentially extending beyond the single season.

Years	1–2	3	4–6	7–10	>10	∑
Number of Colonies	16	28	19	12	3	78

## Data Availability

Data used in the submitted manuscript can be made available to a limited degree after a reasonable request to the corresponding author. Personal data of citizen scientists/reporters or the location–exact addresses will not be available due to violation of privacy.

## Supplementary Materials

The following are available online at https://www.mdpi.com/article/10.3390/insects12121127/s1, Table S1: Overview of all received reports. Table S2: Outcome of citizens/reporter’s interviews. Table S3: Summary of reports by height of nesting/swarming sites. Table S4: Summary of reports by nesting/swarming site types. Table S5: Comparison of ‘lifestyle’ features: key differences between feral/wild and managed honey bee colonies. Figure S1: Distribution of georeferenced unmanaged bee colonies and swarms: (a) nesting colonies, (b) swarms, (c) ambiguous or unspecified reports. Figure S2: Distribution of human population density of Belgrade by ‘local communities’ units, overlayed with distribution of feral honey bee units.
