# Supplementary material for: Unprecedented Density and Persistence of Feral Honey Bees in Urban Environments of a Large SE-European City (Belgrade, Serbia)
Source: Insects. 2021 Dec 16;12(12):1127. doi: 10.3390/insects12121127 (PMC8706874; doi:10.3390/insects12121127)
Supplement: Supplementary file 1 [file insects-12-01127-s001.zip › insects-1475359-supplementary.pdf]

Supplementary Materials 1

Table S1. Overview of received reports.

Table S2. Outcome of citizens/reporter's interviews.

Table S3. Summary of reports by height.

Table S4. Summary of reports by nesting/swarming site.

Table S5. Comparison of 'lifestyle' features: key differences between feral/wild and managed honey bee colonies.

Figure S1. Distribution of reported unmanaged bee colonies and swarms: (a) nesting colonies, (b) swarms, (c) ambiguous or unspecified reports.

Figure S2. Distribution of human population density of Belgrade by 'local communities' units, overlayed with distribution of reported feral honey bee units.

Table S1. Overview of received reports. When duplicated reports (124) were subtracted there were 1371 reports left. Most of the reports were georeferenced, all except the ones whose location was not possible to retrieve (56). In total 1315 locations/reports were georeferenced.

total number of calls	1745
number of reports of other insects	250
number of reports of honey bees	1495
number of duplicated reports	124
number of reports of honey bees without duplicates	1371
number of reports without address or location	56
number of georeferenced reports	1315

Table S2. Outcome of citizens/reporter's interviews.

**Number of citizen scientist/attempted communication: 542**					
Failed 171		Reached, but useless information 53		Reached, useful information 318	
Phone number nonexistent anymore	88	Unreliable info, lack of understanding of the object reported	34	Very interested in the topic, willing to cooperate, volunteer in the future	93
No one answers the phone or replying to SMS messages	32	Person who reported is not available	7	Neutral toward future cooperation, uninterested in the topic	222
Wrongly written/noted phone number in original data set	29	Does not remember the details	5	Extremely unpleasant	3
Voicemail/ unavailable phone number	22	Reluctant to share details/afraid to share	5		
		Person cannot hear well	2		

Table S3. Summary of reports by height

Category height	Number of reports	Colony	Swarm	Ambiguous
0–1 m	22	1	21	-
1–3 m + ground floor	116	7	94	15
3–9 m, floor I+II	158	35	110	13
9–15 m, floor III+IV	75	32	31	12
15–21 m, floor V+VI	11	9	2	-
>21 m, floor VII+	9	6	3	-
Σ	391	90	261	40

Table S4. Summary of reports by nesting/swarming site

Nesting/swarming site	Swarm	Colony	Ambiguous/ unspecified reports
private garden	5	-	-
on the tree	277	20	42
in the hollow tree	-	18	-
on the ground	11	-	-
on the building/house	54	1	67
chimney	4	32	21
inside the wall/façade	-	182	1
wall (imprecise)	4	-	40
air conditioner	4	7	7
house roof/attic	2	14	5
gutter	2	2	1
balcony	11	4	-
window	11	-	6
wooden window shutter box	61	134	96
ventilation drain	1	7	7
electric pole	1	5	-
Other	15	11	6

Table S5. Comparison of 'lifestyle' features: a review of key differences in life history traits and living conditions between free-living (feral or wild) honey bee colonies and managed honey bees (compiled from: [1-3].

FERAL/WILD HONEY BEES	MANAGED HONEY BEES
free to swarm	swarming prevented (controlled) by beekeeper
prefer smaller nest cavities (40 L)	live in large hives (100 L)
colonies distant from each other	colonies clustered, close proximity to each other
primarily vertical transmission of pathogens and parasites	dominance of horizontal transmission of pathogens and parasites
heterogeneous floral diet	mainly uniform floral diet
no artificial (sugar) feeding	regular artificial supplementary feeding with sugar solutions
natural selection pressures present (selection of traits that increase survival, pathogen tolerance etc.)	human mediated selective breeding (selection of traits that are economically beneficial to a beekeeper
rarely disturbed	regular disturbance (through beekeeping activities, honey extraction, beeswax removing, parasite checks)
no treatments (chemical or antibiotic)	chemical treatments against diseases

Apparently, there are several important differences in 'lifestyle' features and related living conditions between feral and managed honey bee colonies. In various ways, these may generate critical differences in susceptibility to *Varroa* mite [1,2].

Essentially, while feral bees are free to swarm, managed ones are usually prevented by a beekeeper [4]. Generally swarming brings about numerous favorable circumstances, which makes the colonies more tolerant to *Varroa* infestation. First, there is no reproduction (no new brood, nor egg laying) several days before and several weeks after the swarming [4-6]. Consequently, there is also no reproduction for *Varroa* mites during this period, since mite females lay eggs only in bee brood cells [7]. Additionally, when the queen and nearly half of workers leave the old colony by swarming, they take away numerous adult *Varroa* mites on their bodies and thereby reduce the load of mites in the old hive [4,5]. Loftus et al. [4] further noted that bees are more susceptible to *Varroa* mites if they live in large hives than in smaller ones. Since free-living honey bees prefer smaller nest cavities (around 40 L) [5,8] these are filled faster and thus promote frequent swarming [5]. Smaller nests also mean less intensive brood rearing, which consequently reduces *Varroa* reproduction [4]. In contrast, beekeeping practices favor larger hives (>100 L of volume) in which bees can make large stores of honey [4].

Another important difference between managed and feral/wild honey bees is mutual proximity of colonies. In apiaries hives are closely clustered [5], while in naturally occurring colonies they are scattered, more spaced [7]. Consequently, the horizontal transmission of parasites and pathogens, which happens through robbing and drifting in apiaries, is insignificant among the feral colonies [5,7]. Fries and Camazine [9] showed that horizontal transmission of pathogens may be very harmful for the host. The vertical transmission, on the other hand, may lead to less virulent forms of pathogens [10].

Diet and available floral resources make another set of lifestyle differences. Managed bees are often moved (in migratory beekeeping) and offered mainly a monocultural, uniform diet, which results in weakening of individual bee's health and decrease in overall colony health [11]. In addition, managed bees are regularly fed with artificial sugar solutions, which have negative side effects [12].

The relocation of the combs between colonies enables and intensifies horizontal pathogen transmission [9], while regular honey harvest brings frequent disturbances through opening of the hive [13]. On the contrary, feral bees are not exposed to these kinds of stressors.

Recent study [14] on heat transfer differences between man-made nest enclosures and natural cavities showed that hive attributes have significant effect on temperature and humidity inside and hence energy spent on maintaining preferable levels of these two factors; consequently, characteristics of the hive affects behavior and health of the bees.

References:

1. Powell, J. Learning from wild bees and tree beekeeping [The Beekeepers Quarterly, issue 123]. Available online: https://www.naturalbeekeepingtrust.org/learning-from-wild-bees-trees (accessed on 30 October).
2. Kohl, P.L.; Rutschmann, B. The neglected bee trees: European beech forests as a home for feral honey bee colonies. *PeerJ* **2018**, *6*, e4602, doi:10.7717/peerj.4602.
3. Seeley, T.D. *The lives of bees: the untold story of the honey bee in the wild.*; Princeton University Press: 2019.
4. Loftus, J.C.; Smith, M.L.; Seeley, T.D. How honey bee colonies survive in the wild: testing the importance of small nests and frequent swarming. *PLoS ONE* **2016**, *11*, e0150362, doi:10.1371/journal.pone.0150362.
5. Seeley, T.D.; Smith, M.L. Crowding honeybee colonies in apiaries can increase their vulnerability to the deadly ectoparasite *Varroa destructor*. *Apidologie* **2015**, *46*, 716–727, doi:10.1007/s13592-015-0361-2.
6. Seeley, T.D.; Tarpy, D.R.; Griffin, S.R.; Carcione, A.; Delaney, D.A. A survivor population of wild colonies of European honeybees in the northeastern United States: investigating its genetic structure. *Apidologie* **2015**, *46*, 654–666, doi:10.1007/s13592-015-0355-0.
7. Fries, I.; Hansen, H.; Imdorf, A.; Rosenkranz, P. Swarming in honey bees (*Apis mellifera*) and *Varroa destructor* population development in Sweden. *Apidologie* **2003**, *34*, 389–397, doi:10.1051/apido:2003032.
8. Rinderer, T.E.; Tucker, K.W.; Collins, A.M. Nest cavity selection by swarms of European and Africanized honeybees. *J. Apic. Res.* **1982**, *21*, 98–103, doi:10.1080/00218839.1982.11100522.
9. Fries, I.; Camazine, S. Implications of horizontal and vertical pathogen transmission for honey bee epidemiology. *Apidologie* **2001**, *32*, 199–214, doi:10.1051/apido:2001122.
10. Lipsitch, M.; Siller, S.; Nowak, M.A. The evolution of virulence in pathogens with vertical and horizontal transmission. *Evolution* **1996**, *50*, 1729–1741 doi:10.1111/j.1558-5646.1996.tb03560.x.
11. Somme, L.; Moquet, L.; Quinet, M.; Vanderplanck, M.; Michez, D.; Lognay, G.; Jacquemart, A.-L. Food in a row: urban trees offer valuable floral resources to pollinating insects. *Urban Ecosyst* **2016**, *19*, 1149–1161, doi:10.1007/s11252-016-0555-z.
12. Johnson, R.M.; Mao, W.; Pollock, H.S.; Niu, G.; Schuler, M.A.; Berenbaum, M.R. Ecologically appropriate xenobiotics induce cytochrome P450s in *Apis mellifera*. *PLoS ONE* **2012**, *7*, e31051, doi:10.1371/journal.pone.0031051.

13. Büchler, R.; Berg, S.; Le Conte, Y. Breeding for resistance to *Varroa destructor* in Europe. *Apidologie* **2010**, *41*, 393–408, doi:10.1051/apido/2010011.
14. Mitchell, D. Ratios of colony mass to thermal conductance of tree and man-made nest enclosures of *Apis mellifera*: implications for survival, clustering, humidity regulation and *Varroa destructor*. *Int. J. Biometeorol.* **2016**, *60*, 629–638, doi:10.1007/s00484-015-1057-z.

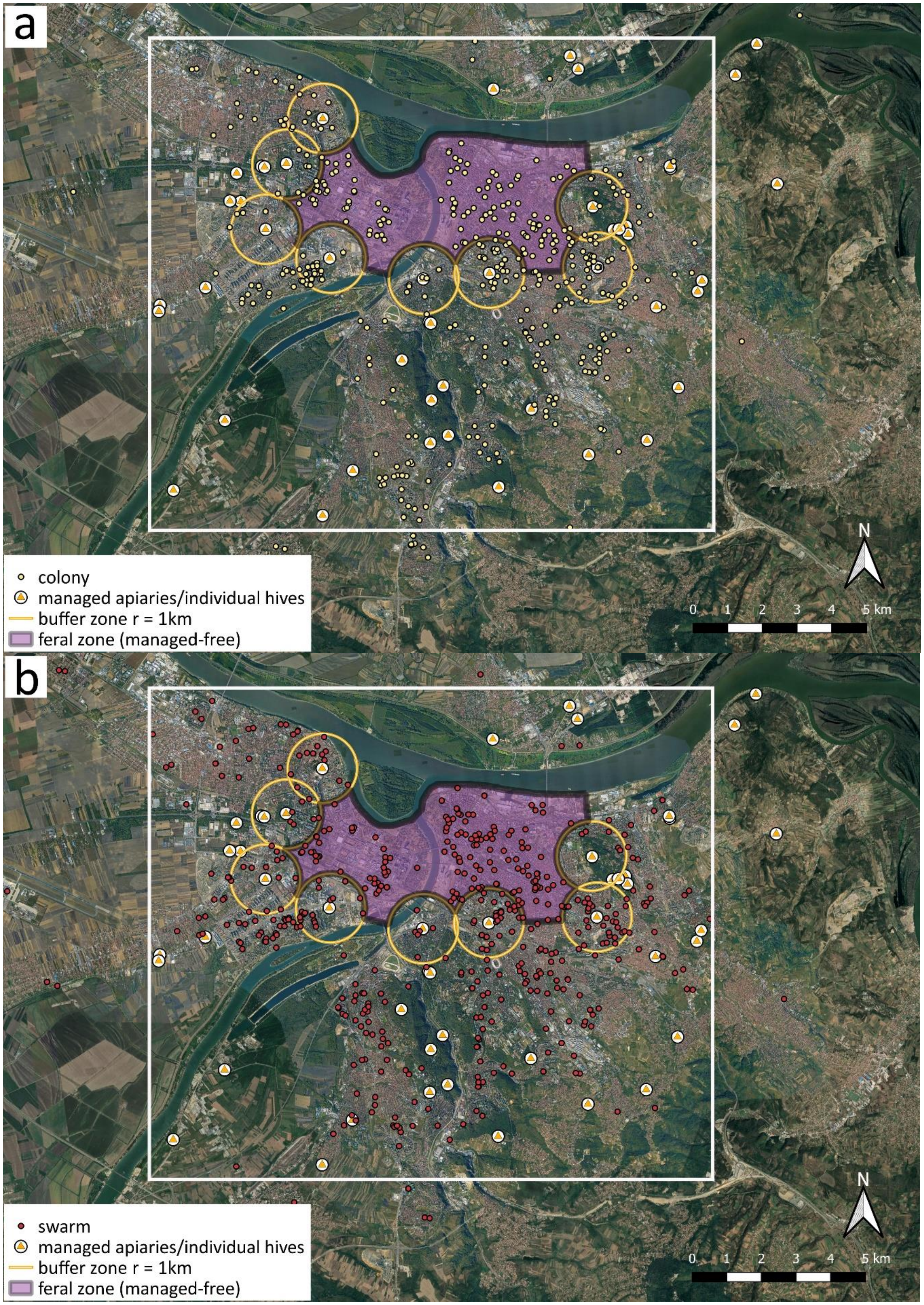
a
colony
managed apiaries/individual hives
buffer zone r = 1km
feral zone (managed-free)
N
0 1 2 3 4 5 km
b
swarm
managed apiaries/individual hives
buffer zone r = 1km
feral zone (managed-free)
N
0 1 2 3 4 5 km

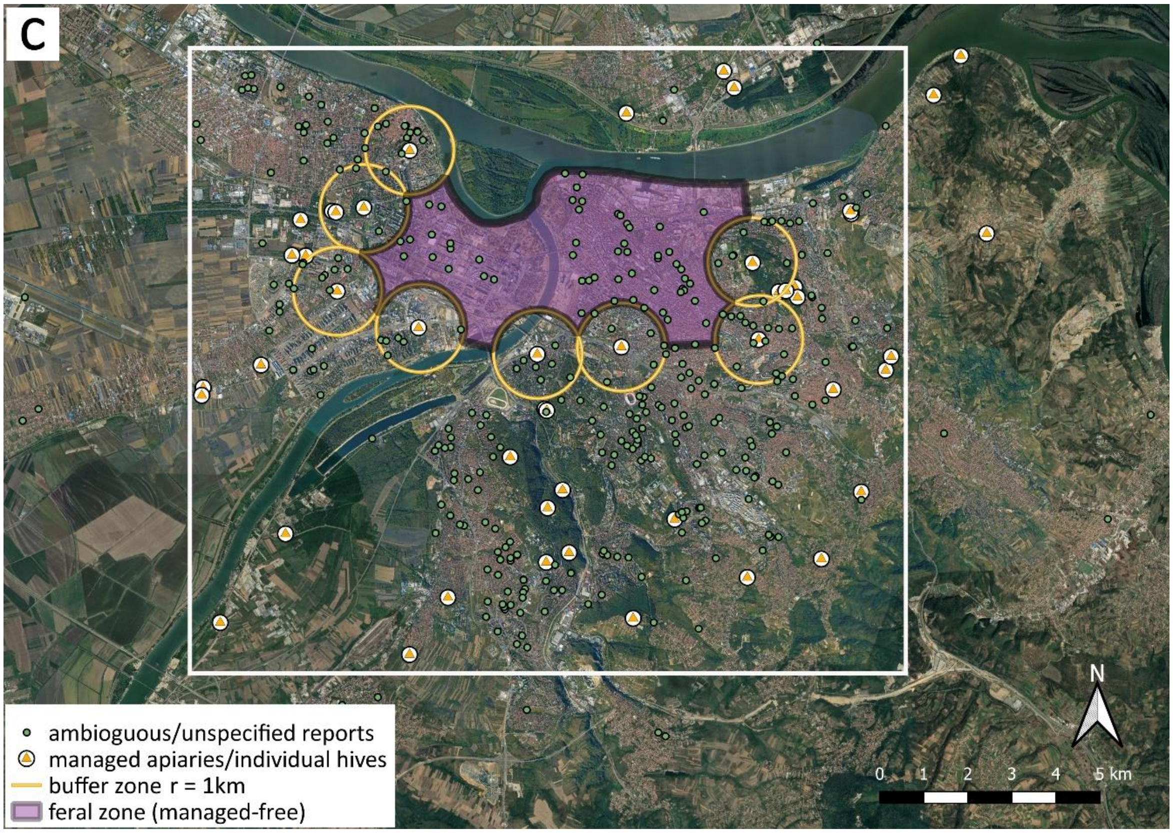

Figure S1. Distribution of georeferenced unmanaged bee colonies and swarms, reported in the period 2011–2017 within the wider Belgrade area (24 more remote locations are not shown, being too widely scattered beyond the coverage of this map): a) nesting colonies, b) swarms, c) ambiguous or unspecified reports (either a colony or a swarm). The locations of known managed apiaries (or individual hives) are also shown, with the particular focus on those which surround the urban core area (each shown with circular 'buffer zone' of r = 1 km). The urban core is presumed to harbor mostly the self-sustained feral bee colonies, and consequently, the swarms produced mostly by them; accordingly, we delimited a tentative 'feral zone' (or 'managed-free zone'). The white rectangle (ca. 16.2 × 14.4 km) delimits the area analyzed in more detail – compare the Figures 6–8. (These maps are also available as separate high-resolution images, upon request to J.B.D.)

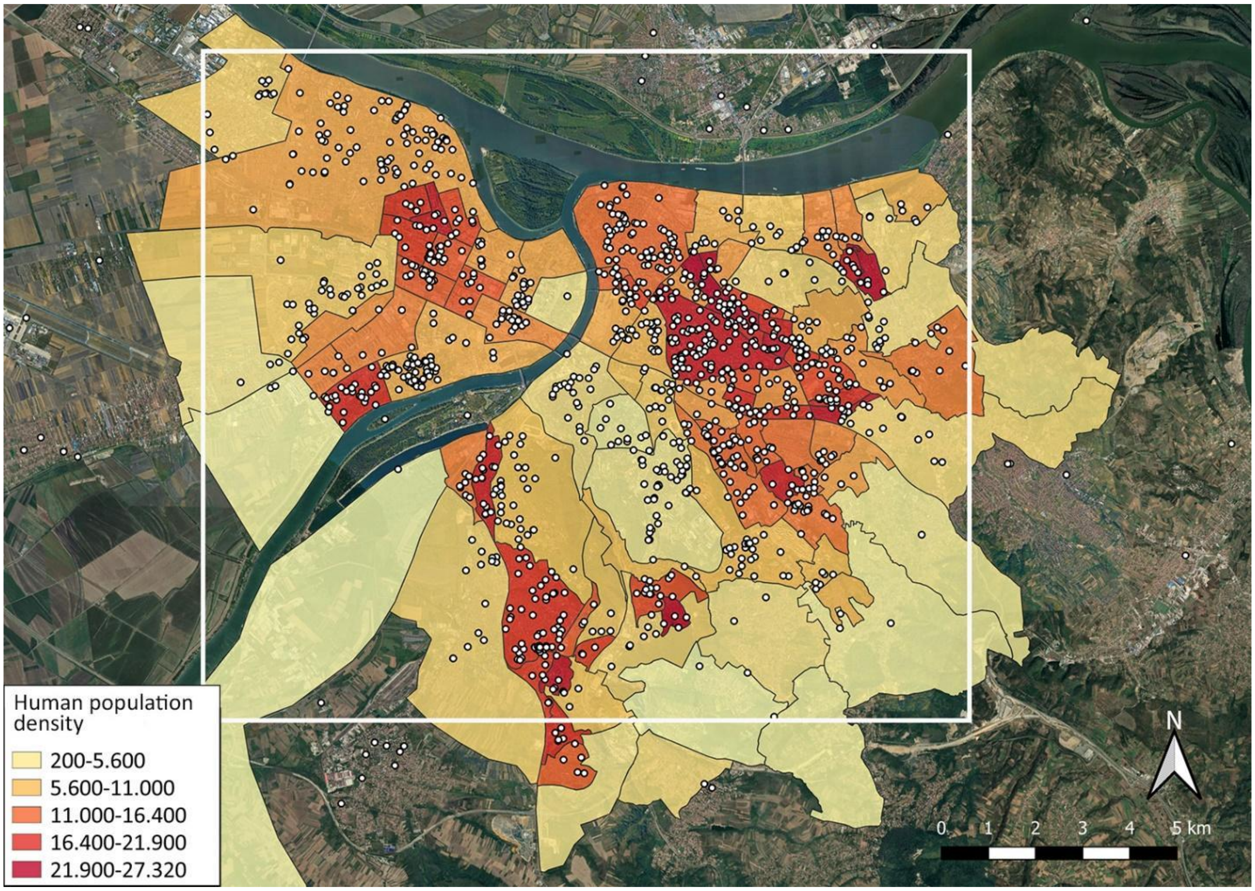

Figure S2. Distribution of human population density (per square kilometer) of Belgrade municipalities is shown by lowest available administrative units ('local communities'; data from the latest census – of 2011); only the units included in the analysis are shown. Distribution of reported occurrences of feral honey bee units within the same area is shown combined (colony+swarm+unknown), localities not overlapping with census units were not used in analysis. White rectangle denotes the same area as in other maps (Figures 2, 6–8, S1).
